# Insights Into Pathogen Diversity and Antimicrobial Resistance Profiles in the Intensive Care Units of a Regional Hospital in Oman

**DOI:** 10.7759/cureus.81014

**Published:** 2025-03-22

**Authors:** Faisal Al Sawafi, Hasan Aldwaha, Houda Almusalhi, Khalid Alnairi, Saleh Alshukairi, Raiya AlHabsi, Badr Alrashdi, Yogesh Manhas, Yassir Hamadalnil, Abhijit Nair

**Affiliations:** 1 Critical Care, Ibra Hospital, Ibra, OMN; 2 Medicine, Ibra Hospital, Ibra, OMN; 3 Anesthesiology, Ibra Hospital, Ibra, OMN; 4 Epidemiology and Public Health, Ibra Hospital, Ibra, OMN; 5 Microbiology, Ibra Hospital, Ibra, OMN; 6 Pediatrics, Ibra Hospital, Ibra, OMN; 7 Laboratory Medicine, Ibra Hospital, Ibra, OMN

**Keywords:** anti-microbial resistance, gram-negative bacteria (gnb), gram-positive bacteria, hospital-acquired infections, intensive care unit

## Abstract

Background: Hospital-acquired infections (HAIs), also known as nosocomial infections, are considered one of the greatest challenges to hospitals and the healthcare system, especially in intensive care units (ICUs). The biggest challenge comes from developing antimicrobial resistance (AMR), which is associated with an increased risk of morbidity and mortality. A regularly updated antibiogram is essential for the ICU physician to assist in choosing empirical antibiotics appropriately till culture results are released.

Methods: We conducted a retrospective study to determine the spectrum of AMR among bacteria encountered in the ICU. We aim to determine the prevalence and distribution of resistant strains.

Results: The study found a total of 498 samples that were positive for pathogens. Gram-negative pathogens were the most commonly isolated ones, and *Klebsiella *was the most frequent finding. One hundred and thirty-six (27.3%) isolates were multidrug-resistant organisms (MDRO). *Acinetobacter *was the most common MDRO isolated, followed by carbapenem-resistant *Enterobacteriaceae *(CRE). Gram-negative pathogens were sensitive to aminoglycosides, except *Acinetobacter*, which was sensitive to cotrimoxazole and colistin. Carbapenem-resistant *Enterobacteriaceae *were the most difficult to treat MDRO.

Conclusion: The development of an antibiogram for each health institute can help in effectively managing and treating nosocomial infections and reduce the risk of antimicrobial infections. It will guide the physician and help avoid the misuse of antibiotics.

## Introduction

Hospital-acquired infections (HAIs), also known as nosocomial infections, are infections that are typically acquired after 48 hours of hospitalization [1]. These infections exert significant pressure on healthcare systems, particularly within intensive care units (ICUs), where patients are often immunocompromised and highly susceptible to infections [2]. Intensive care units are recognized as high-prevalence areas for HAIs, contributing substantially to increased morbidity, mortality, and healthcare costs [3]. The risk of acquiring HAIs is influenced by various factors, including the effectiveness of a facility’s infection control policies, the patient’s immune status, and the prevalence of pathogens within the community. Specific risk factors for HAIs in ICU settings include immunosuppression, advanced age, prolonged hospital stays, underlying comorbidities, frequent healthcare interactions, mechanical ventilatory support, recent invasive surgeries, and the use of indwelling devices [4, 5]. Intensive care units are often considered the epicenter for the development of antimicrobial resistance (AMR), largely due to the critical nature of illnesses treated, the widespread use of invasive devices (such as endotracheal tubes and vascular and urinary catheters), and the extensive and sometimes inappropriate use of antibiotics [6]. The variable adherence to infection control practices further exacerbates this challenge. Consequently, the management of infections within the ICU remains a growing challenge. Intensive care unit physicians must rely on regularly updated antibiograms to guide empirical antibiotic therapy effectively while awaiting culture results [7]. This study aims to determine and examine the spectrum of antimicrobial resistance among bacteria encountered in the ICU with a focus on understanding the prevalence and distribution of resistant strains. The findings will provide valuable insights into the local epidemiology of HAIs and support the development of more effective infection control strategies tailored to this setting.

## Materials and methods

This study was conducted at Ibra Hospital in Ibra, which is a secondary care hospital under the Ministry of Health, Sultanate of Oman, that serves North Sharqiya Governorate. The study proposal was approved by the Research and Studies Committee of the Directorate General of Health Services at North Sharqiya Governorate (proposal ID: MoH/CSR/24/27924). This retrospective analytical record-based cross-sectional study was carried out for data collected from 1^st^ January 2022 to 30^th^ June 2023 with a total of 246 clinical isolates obtained from various clinical samples from different ICUs in the hospital. Its ICU includes 10 beds. All the patients in these ICUs were suffering from signs and symptoms of infection during the study period.

Study design and sample size

Two hundred and forty-six clinical isolates were collected from various clinical specimens, including blood, respiratory secretions, urine, and wound swabs. The study included all microbiological isolates obtained from ICU patients during the study period. We used convenience sampling, which included all the isolates during the defined study period. We included all bacterial isolates from clinical samples of ICU-admitted patients. We analyzed only the first identified isolate per patient with verified final results. We excluded all duplicate isolates from the same patients. We also excluded fungal or viral pathogens, surveillance cultures, contaminated samples, and those reported with intermediate sensitivity.

Patients were diagnosed based on clinical presentation, and they were subjected to full clinical history taking with a focus on associated risk factors such as hospital stay duration, underlying medical conditions, and invasive medical procedures. The diagnostic criteria and all investigations were performed following relevant local guidelines, protocols, and regulations, and all the data were extracted from the laboratory information system (LIS). Identification was based on colonial morphology, gram stain, and standard biochemical tests. According to the Clinical and Laboratory Standard Institute (CLSI), the antimicrobial susceptibility of isolated organisms was detected using the Kirby-Bauer disc diffusion method [8]. Blood cultures were done using an automated BACT/ALERT 3D microbial detection system (bioMérieux Inc., Durham, NC, USA) and incubated for five to seven days. Positive blood culture bottles and other isolated samples were initially grown on blood, MacConkey, and chocolate agars for 24 to 48 hours at 37°C. The collected data were entered into Microsoft Excel 2024 (Microsoft Corp., Redmond, WA, USA), and expressed as absolute numbers and percentages.

## Results

**Figure 1:**
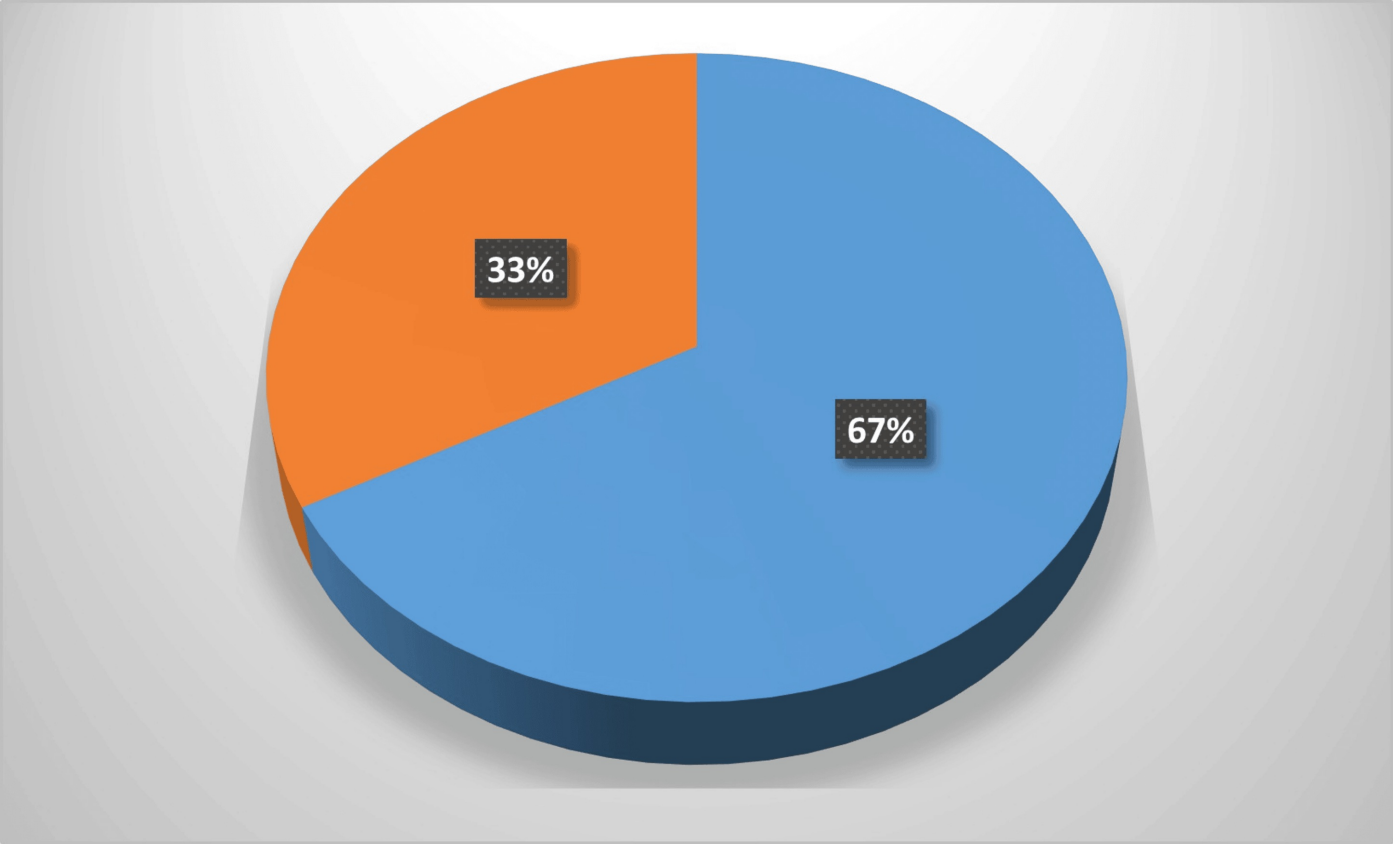
Incidence of microorganisms in the ICU Blue colour: Gram-negative, Orange colour: Gram-positive

The total number of samples that grew pathogens was 498 (Table 1). Endotracheal samples had the highest positivity rate (178, 35.7%), followed by urine samples (86, 17.26%) and blood cultures (66, 13.25%). The majority of the isolated pathogens were Gram-negative bacilli (334, 67%) followed by Gram-positive bacilli (164, 33%) (Figure 1).

**Table 1:** Prevalence of positive samples in ICUs CSF: cerebrospinal fluid

Clinical samples	Total	Prevalence (%)
Blood culture	66	13
Catheter tip culture	33	7
Corneal scraping culture	1	0
Ear swab culture	1	0
Endotracheal secretion culture	178	36
Eye swab culture	7	1
Pediatric blood culture	19	4
Pleural fluid culture	4	1
Sputum culture	41	8
Swab culture	12	2
Urine culture	86	17
Wound culture	37	7
Abscess/pus culture	9	2
Nasal swab culture	3	1
CSF culture	1	0
Total	498	100

Gram-negative pathogens

The various Gram-negative pathogens isolated were *Pseudomonas aeruginosa*, *Klebsiella pneumoniae*, *Acinetobacter baumannii*, *Citrobacter koseri*, *Escherichia coli*, *Enterobacter cloacae*, *Proteus mirabilis*, and *Serratia marcescens*. Among Gram-negative pathogens, *Klebsiella *was the most frequently isolated pathogen (110, 22%), followed by *Pseudomonas aeruginosa* (95, 19%), *Escherichia coli* (59, 12%), and *Acinetobacter baumannii (*48, 10%).

Gram-positive pathogens

The various Gram-positive pathogens isolated were *Staphylococcus aureus*, *Enterococcus faecium*, *Enterococcus faecalis*, *Streptococcus*, and other *Staphylococcus*. Other staphylococci were 58 (12%), and *Staphylococcus aureus* was 50 (10%). These were the most common Gram-positive pathogens isolated, followed by *Streptococcus (*30, 6%). One hundred and thirty-six (27.3%) isolates were found to be multidrug-resistant (Table 2). Multidrug-resistant organisms (MDRO) isolate count incidence is summarized in Table 3.

**Table 2:** Incidence of isolated microorganisms in the ICUs

Parameters	Microorganisms	Number	Incidence (%)
Gram-negative	Pseudomonas aeruginosa	95	19
	Klebsiella pneumoniae	110	22
	Acinetobacter baumannii	48	10
	Citrobacter koseri	4	1
	Escherichia coli	59	12
	Enterobacter cloacae	8	2
	Proteus mirabilis	5	1
	Serratia marcescens	5	1
Gram-positive	Staphylococcus aureus	50	10
	Other *Staphylococcus*	58	12
	Enterococcus faecium	20	4
	Enterococcus faecalis	6	1
	Streptococcus	30	6
	Total	498	100

**Table 3:** MDRO isolate count incidence MRSA: methicillin-resistant *Staphylococcus aureus*; VRE: vancomycin-resistant enterococci; ESBL: extended-spectrum beta-lactamases; CRE: carbapenem-resistant *Enterobacteriaceae*

Multidrug-resistant organisms (MDRO)	Isolated count	Incidence (%)
MRSA	18	13
VRE	5	4
ESBL	27	20
CRE	33	24
Acinetobacter	39	29
Pseudomonas	14	10
Total	136	100

Sensitivity pattern

Among routinely tested antibiotics, aminoglycosides retained sensitivity against Gram-negative pathogens (80% to 90%). For Gram-positive pathogens, vancomycin, linezolid, clindamycin, and macrolides had 80% sensitivity. Streptococcus sensitivity to penicillin and erythromycin was found to be 77% and 70%, respectively.

Sensitivity pattern of MDROs

Carbapenem-resistant *Enterobacteriaceae *(CRE) were the most difficult-to-treat organisms. We found tetracyclines to be 62% sensitive against CRE, tigecycline 50%, gentamicin 32%, and colistin 20% sensitive. Tetracyclines and tigecycline were not among the routinely tested antibiotics and were tested only three and two times, respectively, out of 33 isolates of CRE in this study. *Pseudomonas *was found to be 56% sensitive to amikacin, 40% to colistin, and 36% to piperacillin-tazobactam. *Acinetobacter *had a 51% sensitivity to trimethoprim-sulfamethoxazole, whereas sensitivity to colistin was 38% sensitive. There was a 90% sensitivity of carbapenems for the extended-spectrum beta-lactamases (ESBLs). Vancomycin and linezolid had good sensitivity (100%) against methicillin-resistant *Staphylococcus *(MRSA). Similarly, linezolid has good activity against vancomycin-resistant enterococci (VRE).

## Discussion

Patients in the ICU are at a higher risk of HAIs owing to the following risk factors: advanced age, immunosuppression, prolonged admissions, invasive procedures, and use of medical devices such as intravascular catheters, endotracheal tubes, and urine catheters. Along with the widespread use of antimicrobials, these factors provide a suitable environment for bacteria to grow and acquire resistance [9].

In our study, *Staphylococcus aureus* accounted for 10% of the isolates, which differs from a study done in Ethiopia (26%) and also differs from a study done in an ICU in India in which it was only 2% [10,11]. The reason for this may be due to the differences in antimicrobial policies, infection control measures taken, and distribution of resistance genes [12]. Out of these, 36% of the Gram-positive were MRSA. *Klebsiella pneumoniae* was the most frequently isolated Gram-negative pathogen (22%). *Klebsiella pneumoniae* is normally part of the gastrointestinal tract (GIT) flora and carries a high risk of ICU-admitted cases. This pathogen has the remarkable ability to develop antibiotic resistance through various routes, including horizontal gene transfer. *Klebsiella pneumoniae* carbapenemase (KPC), New Delhi metallo-β-lactamase (NDM), and oxacillinase-48 (OXA-48) can cause carbapenem resistance. The establishment of resistant strains is associated with poor clinical outcomes, morbidity, longer hospital stays, fewer treatment alternatives, and high mortality [13]. The other frequently isolated Gram-negative pathogens were *Pseudomonas aeruginosa* (19%), *Escherichia coli* (12%), and *Acinetobacter baumannii* (10%). This differs from a study done in Saudi Arabia in which the Gram-negative prevalence is as follows: *Acinetobacter baumannii*, followed by *Pseudomonas aeruginosa*, *Escherichia coli*, *Klebsiella pneumoniae*, *Stenotrophomonas maltophilia*, and *Enterobacter *[14]. Magill et al. conducted a survey of 183 hospitals in the USA and found that, of 11,282 patients, 452 experienced at least one infection associated with medical care [15]. Of the 504 confirmed infections, the most prevalent were gastrointestinal infections (17.1%), surgical site infections (21.8%), and pneumonia (21.8%). The most frequently reported pathogen was *Clostridium difficile*, accounting for 12.1% of infections linked to healthcare. Of these patients, 25.6% had device-associated infections (ventilator-associated pneumonia, urinary tract infection, and central line-associated bloodstream infections) [15].

Tarek et al. collected surveillance data on non-hospitalized Egyptian patients with urinary tract infections (UTIs) to develop strategies against multidrug-resistant pathogens [16]. Of the 15,252 samples collected from all the patients, 61% were positive cultures. Of those, female patients accounted for 67.5% of the samples, with infants and elderly patients showing the highest positive cultures (74.4% and 69.2%, respectively). The analysis revealed that *Escherichia coli *was the most frequently identified bacterium in our isolates and *Klebsiella *spp. displayed high resistance to the majority of tested antibiotics. Trimethoprim/sulfamethoxazole showed a significantly increased sensitivity, especially against *Escherichia coli *[16]. In this study, 86 (17.26%) of the urine samples were positive.

In a retrospective cross-sectional study conducted between January 2016 and December 2019, Duan et al. identified 4,161 positive culture samples from 18,798 different specimens [17]. Of these, 9,645 were respiratory tract samples, and 9,153 were blood samples from patients with lower respiratory tract infections (LRTIs) that were analyzed for pathogen incidence and antibiotic sensitivity. Gram-negative bacterial strains were more common than Gram-positive bacterial strains in respiratory tract cultures. The most prevalent bacterium in the pediatric ward was *Staphylococcus aureus* (19.19%), whereas *Pseudomonas aeruginosa* was the predominant pathogen in the adult respiratory ward (21.49%) and the respiratory intensive care unit (RICU) (35.67%). Gram-positive bacteria are the predominant microorganisms implicated in LRTIs in blood cultures. The most common pathogens in adult respiratory departments and pediatric hospitals were *Staphylococcus aureus* (21.8%) and *Staphylococcus epidermidis* (47.20%). Nonetheless, the majority of pathogens in the RICU were Gram-negative bacteria, with *Klebsiella pneumoniae* (27.57%) being the most common [17].

In our study, 178 (35.7%) of endotracheal samples were positive, followed by 86 (17.26%) of urine samples and 66 (13.25%) of blood cultures. Culture and sensitivity testing revealed that 136 (27.3%) of the isolates were multidrug-resistant. One of the main causes of HAIs, MRSA, is frequently linked to high rates of morbidity, death, length of hospital stays, and financial burden. Hospital-associated and community-associated infections are two subtypes of MRSA. The reported incidence of MRSA infection ranges from 7% to 60% [18].

Limitations

There are several limitations to this study. This was a single-center study. We did not investigate the overall patient outcomes in terms of length of stay, morbidity, mortality, and the cost of treatment. Thirdly, it was a retrospective study, thus making it prone to various biases like selection and observer bias and also the possibility of missing data. As we did not compare outcomes like mortality, length of hospital/ICU stay, or requirement of mechanical ventilation or vasopressors/inotropes, we could not perform detailed inferential statistics. Lastly, being a retrospective study, there are high chances of confounding, which might not have been addressed.

## Conclusions

Based on the findings of this study, we conclude that developing an antibiogram for every health institute is very important because it can help in the comprehensive management of underlying conditions and thus treat nosocomial infections effectively. This will eventually reduce the risk of AMR. It will also be a guide for the treating physician and thus will avoid the empirical use and overall misuse of antibiotics.
